# The state of current research on COVID-19 and antibiotic use: global implications for antimicrobial resistance

**DOI:** 10.1186/s41043-023-00386-2

**Published:** 2023-05-12

**Authors:** Sa’ed H. Zyoud

**Affiliations:** 1 Department of Clinical and Community Pharmacy, College of Medicine and Health Sciences, An-Najah National University, Nablus, 44839 Palestine; 2 Clinical Research Centre, An-Najah National University Hospital, Nablus, 44839 Palestine

**Keywords:** COVID-19, Antibiotic use, Antimicrobial stewardship, Antimicrobial resistance

## Abstract

**Background:**

During the initial stages of the coronavirus disease 2019 (COVID-19) pandemic, the administration of antibiotics to patients was prevalent in numerous countries. Despite this, the rising threat of antimicrobial resistance (AMR) presents a significant public health concern. The escalation of AMR has been exacerbated by the ongoing COVID-19 pandemic. Against this backdrop, the primary aim of this study was to conduct a bibliometric and visual analysis of research pertaining to the use of antibiotics in COVID-19.

**Methods:**

This study examined documents indexed in Scopus between 2020 and 2022. To visualize the trends and hotspots of research related to antibiotics and COVID-19, as well as collaborative networks, the researcher utilized version 1.6.18 of the VOSviewer software. Scopus data were analysed to extract information on the types of publications, annual research output, countries, institutions, funding agencies, journals, citations, and highly cited references. Microsoft Excel 2019 was used to process and organize the extracted data.

**Results:**

This study analysed 1137 documents related to COVID-19 and antibiotics and found that the number of publications increased from 130 in 2020 to 527 in 2022. These publications included 777 (68.34%) articles and 205 (18.03%) reviews. The top five countries in terms of scientific production were the United States (*n* = 231; 20.32%), the United Kingdom (*n* = 156; 13.72%), China (*n* = 101; 8.88%), India (*n* = 100; 8.8%), and Italy (*n* = 63; 5.54%), and the leading institutions were *Imperial College London* (*n* = 21; 1.85%), *University of Oxford* (*n* = 20; 1.76%), and *University College London* (*n* = 15; 1.32%). The *National Natural Science Foundation of China* provided funding for the highest number of articles (*n* = 48; 4.22%), followed by the *National Institutes of Health* (*n* = 32; 2.81%). The most productive journals were *Antibiotics* (*n* = 90; 7.92%), *Journal of Antimicrobial Chemotherapy* (*n* = 30; 2.64%), and *Infection Control and Hospital Epidemiology* (*n* = 26; 2.29%). Finally, the research hotspots identified in this study were ‘*antimicrobial stewardship during the COVID-19 outbreak*’ and ‘implications* of the COVID-19 pandemic on the emergence of antimicrobial resistance.’*

**Conclusions:**

This is the first bibliometric analysis of COVID-19-related research on antibiotics. Research was carried out in response to global requests to increase the fight against AMR and awareness of the issue. More restrictions on the use of antibiotics are urgently needed from policy makers and authorities, more so than in the current situation.

## Background

Antimicrobial resistance (AMR) has emerged as a critical global public health concern owing to the growing incidence of resistant human pathogens [1, 2]. The primary contributor to the development of AMR is the widespread and inappropriate use of antibiotics [3, 4]. Although antibiotics are not effective in treating coronavirus disease 2019 (COVID-19), a viral illness [5], secondary bacterial infections, such as pneumonia, may ensue in individuals with viral respiratory infections, necessitating the use of antibiotics [6]. This co-pathogenesis of viral and bacterial infections has led to the use of antibiotics in the management of COVID-19 [7]. Furthermore, antibiotics have been frequently prescribed to patients with COVID-19, despite their ineffectiveness against viruses, including the one responsible for COVID-19 [8].

In order to attain the Sustainable Development Goals (SDGs) and ensure the advancement of global health, there is a pressing need for multisectoral action to counter the menace of antimicrobial resistance [9]. The World Health Organization (WHO) has identified AMR as one of the most significant ten public health challenges that humanity currently faces [10]. The excessive use and inappropriate application of antimicrobial agents represent pivotal factors responsible for the emergence of infections that are resistant to conventional treatments [10].

Although many bibliometric studies [11–15] have been published on COVID-19, none of them discuss the link between the illness and antibiotic use. However, since this is one of the most compelling hypotheses about AMR, it is important to identify potential developments that could provide researchers with direction when designing new studies [16]. Furthermore, researchers can also conduct a more in-depth investigation of international collaborations using the bibliometric technique and evaluate the impact that scientific publications have within the research community by determining the most hot topics that concern this area of research [17, 18]. Therefore, this study uses bibliometrics to analyse the relevant information from articles on "COVID-19 and antibiotic use" to improve understanding of the research history and current state of knowledge in this field to explore the research highlights. The significance of this study lies in its examination of the pressing issue of the increased use of antibiotics during the COVID-19 outbreak and its correlation with antimicrobial resistance. The study's findings could potentially aid healthcare providers, regulators, and researchers in formulating strategies to mitigate the impact of AMR and minimize antibiotic use in COVID-19 patients. Additionally, the bibliometric and visual analysis included in the research would provide a comprehensive overview of the research domain, highlighting the most prominent research publications, authors, and institutions involved in this matter. Overall, this study is essential to understand the current state of research and address the challenges posed by the COVID-19 pandemic in the context of AMR.

## Methods

### Study design

Bibliometric methods were used to conduct a descriptive analysis of publications related to COVID-19 and antibiotics using a cross-sectional approach.

### Database used

A comprehensive literature search was conducted on the Scopus database, with no predetermined beginning date, up until December 1, 2022. The decision to utilize the Scopus database for this study was influenced by a number of factors. Scopus is a renowned bibliometric and scientific research database [19–23] that comprises approximately 30,000 of the world's most prestigious journals. It provides a comprehensive summary of research findings from diverse disciplines, such as science, medicine, and technology [24–26]. Scopus provides researchers with sophisticated citation analysis tools, enabling them to monitor and analyze the impact of research publications over time. These instruments can be used to evaluate the quality and significance of research findings as well as to identify critical trends and emerging research areas. Scopus is a user-friendly database with an intuitive interface that makes it simpler for researchers to locate the necessary literature. Furthermore, its advanced search capabilities enable users to narrow down their search criteria and quickly retrieve the most relevant literature.

### Search strategy

In subsequent steps of the search, we used a variety of synonyms for both COVID-19 and antibiotics.

*Step 1* To achieve the objectives of this investigation, the terms that were typed into the Scopus engine were chosen based on the findings of previous scientometric studies on COVID-19 [20, 27–31]. Both the title and the abstract have been updated to contain each of the terms.

*Step 2* After the documents were located in Step 1, the titles of those documents were examined to determine whether they contained the phrase "antibiotics and related phrases". This search term included ‘antibacterial’, ‘antibacterial’, ‘antibiotic’, ‘antimicrobial’, and ‘antimicrobial’. We use the keywords ‘antibiotics’ and ‘antimicrobial’ because we are more interested in antibiotics in general than in particular drugs. Since other search fields, such as Abstract and Keywords, have been widened, the search method for antibiotic-related phrases has been limited to the title alone to improve the accuracy of the results. Numerous publications not related to antibiotics (that is, false positive data) were found if we expanded the search to abstracts and keywords. The findings of the researchers in previous studies [32–34] indicate that the inclusion of search elements in the title, rather than performing a topic search (title, abstract, and keywords), results in a large improvement in specificity with just a slight reduction in sensitivity.

*Step 3* The exclusion criteria entailed the removal of publications that were categorized as erratum. The search for relevant literature was not limited by language.

### Validation of search strategy

The research strategy used in this study was validated to ensure the dependability and accuracy of the results. To validate the absence of false-positive results, the titles and abstracts of documents with even numbers (5, 10, 15, 20, etc.) up to number 300 were evaluated. The research strategy was refined, and false-positive outcomes were eliminated until a completely accurate set of randomly screened outcomes was obtained. Furthermore, the research productivity of ten active authors in the field was examined to validate the absence of false-negative outcomes or missing results. Using the Spearman correlation test, the obtained results were compared to those generated by the research strategy. The study's findings revealed a significant and strong correlation (*p* = 0.001; *r* = 0.955) between the two sets of results, indicating the research strategy's high level of validity. Notably, Sweileh et al. previously used this validation approach [21, 35, 36].

### Data export and bibliometric indicators

The refined results were exported to Microsoft Excel, and the analysis generated five primary bibliometric indicators.

**First,** the retrieved publications were examined to determine the growth trend and research topics related to antibiotics. This evaluation helped clarify the current state of research and highlight the areas that have been investigated thus far.

**Second,** data were analysed to identify the key countries, institutions, journals, and funding organizations that have contributed significantly to COVID-19 and antibiotic research. This analysis provided useful information about prominent researchers and institutions at the forefront of this field.

**Third,** a frequency map of terms used in titles and abstracts was created to identify research themes involved in the development of COVID-19 and antibiotics. This approach helped identify critical issues and research areas in this field.

**Fourth,** an international research partnership map was created to highlight the countries and institutions that have worked together the most on COVID-19 and antibiotic research. This analysis helped identify collaborative research institutions and nations that are leaders in international research partnerships.

**Finally,** the top ten articles cited on COVID-19 and antibiotics were compiled. This analysis helped identify the major research publications in this field and provided insight into the most influential research to date.

### Visualization analysis

Following data extraction, VOSviewer software was used to analyse the data (version 1.6.18). VOSviewer generated visual cooperation network graphs of countries and terms in titles and abstracts. A circle was used to represent each country or term. The distance between the two circles varied depending on the strength of the link term. Different clusters were represented with different colours to determine the hot topics in this field. The size of the circles was related to the frequency with which the terms appeared, and the thickness of the line indicated the strength of the link between the terms [37].

## Results

### Main information

Among the 1137 documents on COVID-19 and antibiotics examined in this study, 130 appeared in 2020, 480 in 2021, and 527 in 2022. A total of 777 (68.34%) of these publications were articles, 205 (18.03%) were reviews and 155 (13.62%) were other types of publication (e.g., letters, editorials).

### Country scientific production

**Fig. 1:**
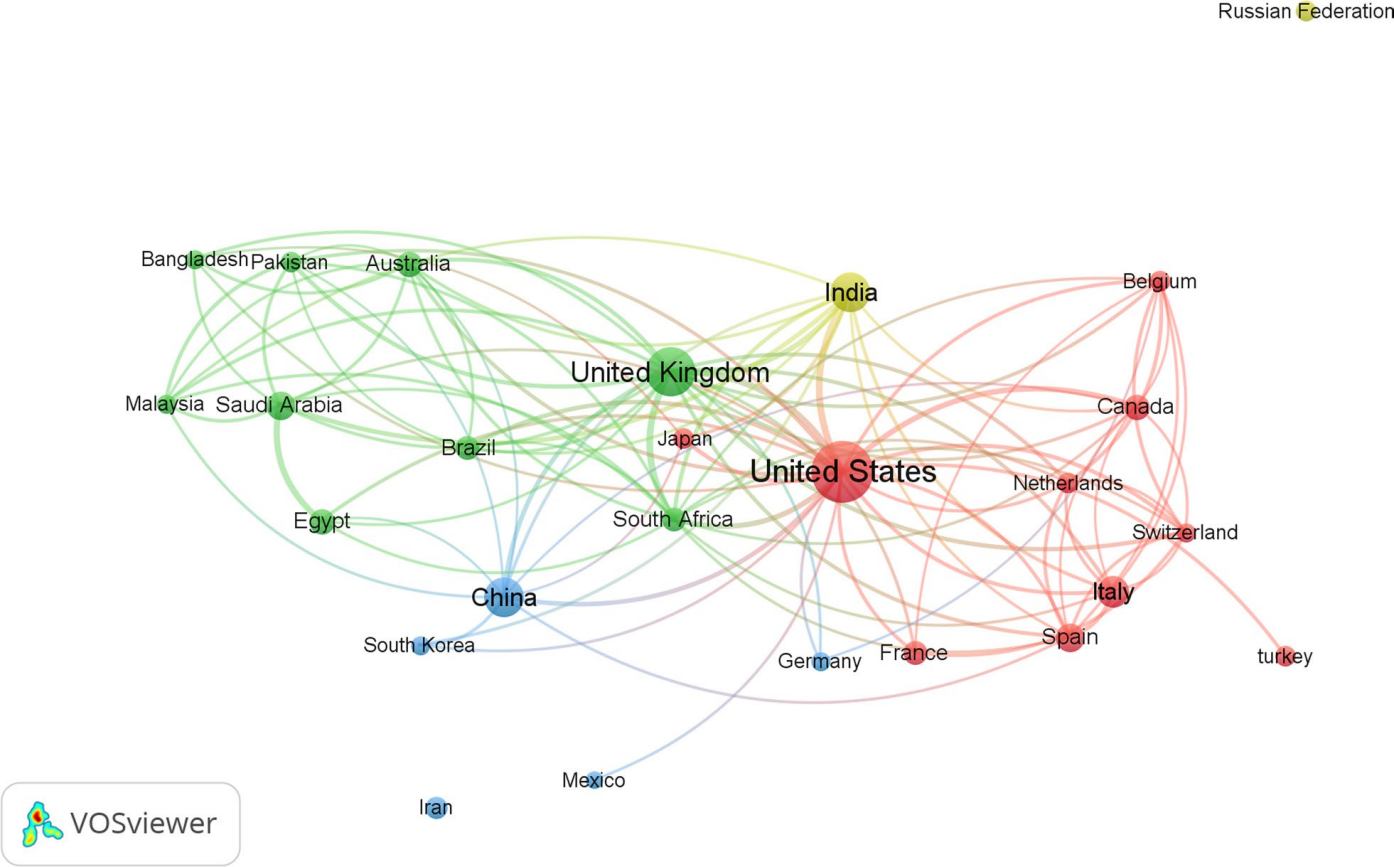
The network visualization map of co authorship collaborations between countries with more than 20 publications

One hundred twenty-six countries/regions were represented among the contributors to the publications on COVID-19 and antibiotics. Table 1 contains a ranking of the ten countries/regions with the highest publications. The United States of America ranked first, with a total of 231 (20.32%) publications, followed by the United Kingdom with 156 (13.72%), China with 101 (8.88%), India with 100 (8.8%), and Italy with 63 (5.54%). The top countries by centrality in collaboration were the USA and the UK (Fig. 1).

**Table 1:** Publications research related to COVID-19 and antibiotic use from the ten most productive countries/regions

Ranking	Country	Number of documents	%
1st	United States	231	20.32
2nd	United Kingdom	156	13.72
3rd	China	101	8.88
4th	India	100	8.80
5th	Italy	63	5.54
6th	Saudi Arabia	53	4.66
7th	Spain	51	4.49
8th	Egypt	41	3.61
9th	Australia	40	3.52
9th	Canada	40	3.52

### Institutional analysis

A total of 4695 institutions were involved in this research area. The top 10 institutions, according to the number of publications, are listed in Table 2. *Imperial College London* (*n* = 21; 1.85%), *University of Oxford* (*n* = 20; 1.76%), *University College London* (*n* = 15; 1.32%), *National Institute of Health Research* (*n* = 14; 1.23%), and *University of Toronto* (*n* = 12; 1.06%) were the top five institutions.

**Table 2:** Publications research related to COVID-19 and antibiotic use from the ten most productive institutions

Ranking	Institute	Country	*n*	%
1st	Imperial College London	UK	21	1.85
2nd	University of Oxford	UK	20	1.76
3rd	University College London	UK	15	1.32
4th	National Institute for Health Research	UK	14	1.23
5th	University of Toronto	Canada	12	1.06
6th	Tehran University of Medical Sciences	Iran	11	0.97
6th	London School of Hygiene & Tropical Medicine	UK	11	0.97
8th	Ministry of Education China	China	10	0.88
8th	The University of Manchester	UK	10	0.88
8th	University of Dundee	Scotland	10	0.88
8th	Cairo University	Egypt	10	0.88
8th	VA Medical Center	USA	10	0.88
8th	Taif University	KSA	10	0.88

### Analysis of funding agencies

The *National Natural Science Foundation of China* provided funding for the highest number of articles (*n* = 48; 4.22%), followed by the *National Institutes of Health* (*n* = 32; 2.81%), and then the *National Institute for Health Research* (*n* = 14; 1.23%); (Table 3).

**Table 3:** The top ten funding agencies with the most publications related to COVID-19 and antibiotic use

Ranking	Funding agencies	Country	No. of publication	%
1st	National Natural Science Foundation of China	China	48	4.22
2nd	National Institutes of Health	USA	32	2.81
3rd	National Institute for Health Research	UK	14	1.23
4th	Department of Science and Technology, Ministry of Science and Technology, India	India	13	1.14
4th	National Key Research and Development Program of China	China	13	1.14
6th	Centers for Disease Control and Prevention	USA	12	1.06
6th	National Science Foundation	USA	12	1.06
6th	Wellcome Trust	UK	12	1.06
9th	Conselho Nacional de Desenvolvimento Científico e Tecnológico	Brazil	11	0.97
9th	European Commission	European Parliament	11	0.97
9th	European Regional Development Fund	European Parliament	11	0.97
9th	National Research Foundation of Korea	South Korea	11	0.97

### Analysis of the journals

The 10 most productive journals on COVID-19 and antibiotic research are listed in Table 4, representing approximately 21.74% of all publications (*n* = 247). *Antibiotics* published the highest number of papers (*n* = 90; 7.92%), followed by the *Journal of Antimicrobial Chemotherapy* (*n* = 30; 2.64%) and *Infection Control and Hospital Epidemiology* (*n* = 26; 2.29%).

**Table 4:** Top 10 productive journals in the “COVID-19 and antibiotics use” area. *Source*: Clarivate, 2022

Ranking	Journal	*n*	%	IF^1^
1st	Antibiotics	90	7.92	5.222
2nd	Journal of Antimicrobial Chemotherapy	30	2.64	5.758
3rd	Infection Control and Hospital Epidemiology	26	2.29	6.520
4th	Journal of Hospital Infection	14	1.23	8.944
5th	International Journal of Molecular Sciences	13	1.14	6.208
6th	Antimicrobial Resistance and Infection Control	12	1.06	6.454
7th	International Journal of Infectious Diseases	11	0.97	12.074
7th	Polymers	11	0.97	4.967
6th	Clinical Microbiology and Infection	10	0.88	13.31
9th	Frontiers in Microbiology	10	0.88	6.064
9th	Science of the Total Environment	10	0.88	10.753
9th	Scientific Reports	10	0.88	4.996

^1^Impact factor (IF) from Journal Citation Reports

### Analysis of highly cited references

The articles most frequently cited related to COVID-19 and antibiotic research are presented in Table 5. The range of the number of citations that made it into the top ten was from 716 to 102 [38–47]. The most cited paper was published by Rawson et al. [42] in *Clinical Infectious Diseases* and was cited 716 times in this field. The articles published by Langford et al. [45] and Huttner et al. [44] ranked second and third, respectively, for the most citations.

**Table 5:** The top 10 articles according to the number of total citations

Authors	Title	Year	Source title	Cited by
Rawson et al. [42]	“Bacterial and Fungal Co-infection in Individuals with Coronavirus: A Rapid Review to Support COVID-19 Antimicrobial Prescribing”	2020	Clinical Infectious Diseases	716
Langford et al. [45]	“Antibiotic prescribing in patients with COVID-19: rapid review and meta-analysis”	2021	Clinical Microbiology and Infection	217
Huttner et al. [44]	“COVID-19: don't neglect antimicrobial stewardship principles!”	2020	Clinical Microbiology and Infection	177
Vaughn et al. [43]	“Empiric Antibacterial Therapy and Community-onset Bacterial Coinfection in Patients Hospitalized with Coronavirus Disease 2019 (COVID-19): A Multi-hospital Cohort Study”	2021	Clinical Infectious Diseases	153
Rawson et al. [47]	“COVID-19 and the potential long-term impact on antimicrobial resistance”	2020	Journal of Antimicrobial Chemotherapy	143
Clancy and Hong Nguyen [41]	“Coronavirus disease 2019, superinfections, and antimicrobial development: What can we expect?”	2020	Clinical Infectious Diseases	141
Getahun et al. [40]	“Tackling antimicrobial resistance in the COVID-19 pandemic”	2020	Bulletin of the World Health Organization	139
Imani et al. [38]	“Antimicrobial nanomaterials and coatings: Current mechanisms and future perspectives to control the spread of viruses including SARS-CoV-2”	2020	ACS Nano	111
Hsu [39]	“How covid-19 is accelerating the threat of antimicrobial resistance”	2020	The BMJ	110
Beovic et al. [46]	“Antibiotic use in patients with COVID-19: A 'snapshot' Infectious Diseases International Research Initiative (ID-IRI) survey”	2020	Journal of Antimicrobial Chemotherapy	102

### Research hotspots

**Fig. 2:**
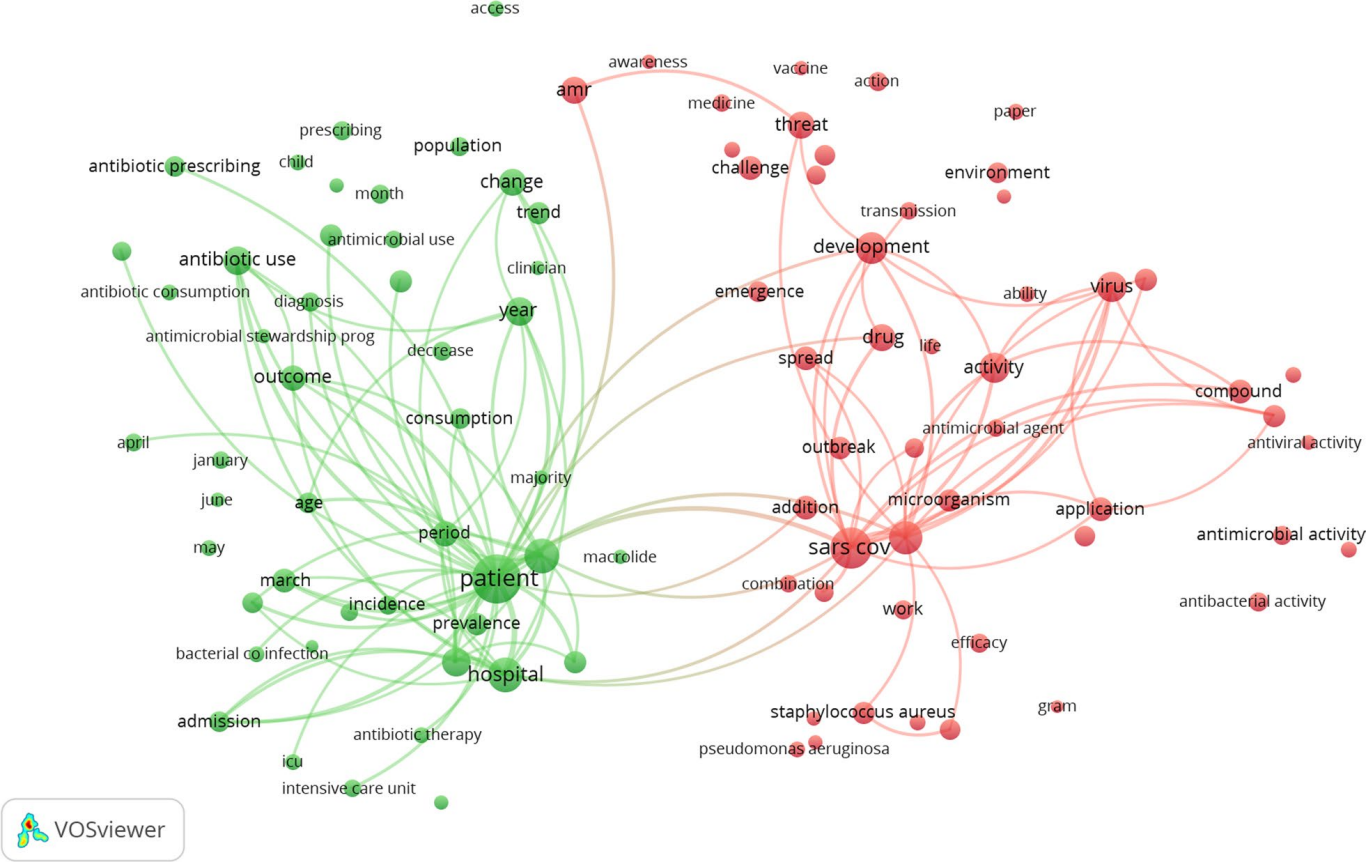
Clustering hot topics by mapping Title/Abstract co-occurrences of terms for COVID-19 research related to antibiotics. Of the 22,087 terms, 162 appeared at least 30 times

Using VOSviewer software, 162 terms that were used more than 30 times in 1137 documents were removed. After that, the cooccurrence network was split up by VOSviewer's clustering function into different colored groups. The more important the terms are, the more likely they are to be close to each other. This makes it easy to learn about the research that is being done. The visual network map shows that all these terms can be put into two clusters (Fig. 2): Cluster 1 (‘*antimicrobial stewardship during the COVID-19 outbreak*,’ green nodes) and Cluster 2 (‘*implications of the COVID-19 pandemic on the emergence of antimicrobial resistance’,* red nodes).

## Discussion

This study represents the first bibliometric investigation into the topic at hand, and provides a comprehensive dataset of the research growth and trends pertaining to this emerging subject matter. Despite the numerous publications on COVID-19 and antibiotics that have emerged in the wake of the COVID-19 epidemic, a more thorough comprehension of the worldwide panorama of COVID-19 and antibiotic research is indispensable.

According to this report, the United States and the United Kingdom are the leading nations in this area. This outcome is not surprising, given that these nations are global leaders in science, including medicine [48]. Our results align with the findings of several previous studies reporting that the United States is the number one country in COVID-19 research [27–29, 49]. According to the current study, China and India ranked third and fourth, respectively, in the number of articles related to COVID-19 and antibiotic research. China is one of the locations around the world where gram-negative bacterial resistance is significant and poses a concern to human and animal health [50]. The key causes of the antimicrobial resistance dilemma are the excessive use of antimicrobials and the unchecked administration of antimicrobials to animals used for food production in China [51]. Furthermore, a recently published scholarly article has proposed that India and China are areas with high levels of AMR in animals [52].

Based on our findings, the *National Natural Science Fund of China*, the *National Institutes of Health*, and the *National Institute for Health Research* were the most productive funding agencies for COVID-19 and antibiotic research. Our data indicate that these three funding agencies have played a crucial role in advancing research on COVID-19 and antibiotic resistance. Notably, the National Natural Science Foundation of China has provided support for a greater number of articles than any other agency, thus demonstrating a significant commitment to research in this field. It is crucial to remember that funding agency rankings can be influenced by a range of factors, including the size of their budget, the breadth of their research aims, and their geographical location [53, 54].

This study identifies the ten most prolific journals in COVID-19 and antibiotic research. Notably, these ten journals account for approximately 21.74 percent of all articles published, a sizeable proportion. Notably, *Antibiotics* has contributed the most articles, demonstrating its leadership in disseminating antibiotic research within the context of COVID-19. This observation implies that there is a significant demand for research in this area, and *Antibiotics* is well-positioned to serve as a forum for researchers to share their findings. The second and third most productive journals in this field, respectively, are the *Journal of Antimicrobial Chemotherapy* and *Infection Control and Hospital Epidemiology*, both of which are concerned with critical aspects of combating the COVID-19 pandemic, such as antimicrobial resistance and infection control. In summary, the findings show that there is a large body of research on COVID-19 and antibiotic use, with several journals emerging as primary sources of information in this field [45, 55]. These findings may be useful for scholars and practitioners who want to stay current on developments in this field.

Based on examining the cooccurrence of terms and the disciplines of research interest indicated, two primary research themes have been identified on COVID-19 and antibiotic research. ‘*Antimicrobial stewardship during the COVID-19 outbreak*’ as a theme was among the main hot topics in the current study. Before the COVID-19 pandemic, AMR rates were steadily increasing throughout the world. There is an escalating apprehension that healthcare systems may be incapable of upholding the implementation of optimal procedures for managing infections and antimicrobial treatments, which could exacerbate the potentiality of antibiotic-resistant microorganisms [56]. A recent meta-analysis that encompassed over 30,000 patients showed that the incidence of bacterial infections in COVID-19 cases was approximately 8.6% [45].

On the contrary, data from a study indicated that 64% of patients were prescribed antibiotics [45]. The potential long-term implications of COVID-19 on antimicrobial stewardship, AMR, and healthcare, in general, are uncertain. Although bacterial coinfections with COVID-19 seem to be infrequent, the utilization of empirical antibiotics remains significant. In light of the COVID-19 pandemic, the WHO has highlighted the need for the integration of antimicrobial stewardship interventions within healthcare systems. Antimicrobial stewardship programmes will be essential to reduce the use of antibiotics when necessary [56]. It is essential to accurately and quickly access diagnostic tools if one wishes to practise successful antimicrobial stewardship. For example, the clinical symptoms of numerous forms of viral respiratory infections are identical to those of bacterial respiratory infections. Due to this, broad-spectrum drugs are often overprescribed or used incorrectly [57, 58].

The current investigation identifies the *implications of the COVID-19 pandemic on the emergence of antimicrobial resistance* as a frequently discussed topic in the context of COVID-19 and antibiotics. The use of antimicrobial agents in COVID-19 patients raises concerns over the development of AMR. The COVID-19 pandemic has exacerbated the development of AMR due to high rates of inappropriate antibiotic prescribing [40]. In addition to avoiding the unnecessary use of antimicrobial agents, effective medical device utilization to mitigate the spread of hospital-acquired infections and adherence to infection prevention and control measures are vital skills required to combat the spread of AMR [59]. Accurate diagnosis of severe COVID-19 with coinfections is also essential. Recent meta-analyses of 23 studies indicate that self-medication with antibiotics, empirical antibiotic administration, and antibiotic prescriptions by general practitioners increase the risk of high levels of AMR during the COVID-19 pandemic [60].

### Recommendations and policy implications of the current study

The significance implications of studying the state of current research on COVID-19 and antibiotic use are multifold.

**First**, understanding the interaction between COVID-19 and antibiotic use is critical to inform clinical practice and management of patients with COVID-19. This is particularly important because antibiotics are often used in the treatment of secondary bacterial infections that can occur in COVID-19 patients.

**Second**, excessive use of antibiotics can lead to the development of antibiotic resistance, which is a major global public health concern. The COVID-19 pandemic has led to an increase in antibiotic use, which could accelerate the emergence and spread of antibiotic-resistant infections. Therefore, studying the current state of research on COVID-19 and antibiotic use can help identify the best practices to manage COVID-19 while minimizing the risk of antibiotic resistance.

**Third**, the COVID-19 pandemic has highlighted the need for a coordinated global response to infectious disease outbreaks. By studying the state of current research on COVID-19 and antibiotic use, we can identify knowledge gaps and areas for further research, which can inform global efforts to combat infectious diseases.

**Finally**, the COVID-19 pandemic has shown that infectious diseases can have profound social and economic impacts, particularly in developing countries. Therefore, understanding the state of the current research on COVID-19 and antibiotic use can help develop effective strategies to mitigate the impact of infectious diseases on vulnerable populations.

### Limitations

Several limitations exist in our study. First, our publications were derived solely from the Scopus database, which may have resulted in insufficient literature. Other databases, such as PubMed and Web of Science, may produce results that vary slightly. Despite this, Scopus is the most popular and widely acknowledged bibliometric analysis database. Second, although two independent reviewers were assigned to evaluate the initial results, there may have been some bias in the publication selection process. Third, only articles published between January 1, 2020, and December 1, 2022, were included in this study; articles published after that date in 2022 were excluded. Consequently, if researchers repeated this study under different conditions, the results could be different.

## Conclusions

The current study is the first bibliometric analysis of COVID-19 research related to antibiotics. The study was carried out in response to calls made on a global scale to intensify the fight against and increase awareness of AMR. The current year has seen a huge surge in publications in this area, indicating that COVID-19 research on antibiotics has attracted much scholarly interest. Several themes, such as ‘antimicrobial stewardship during the COVID-19 outbreak’ and ‘antimicrobial resistance in the COVID-19 landscape’, were the focus of the current literature on COVID-19 and antibiotics. We must now collaborate as a multidisciplinary community to collect data on these changes and collaboratively solve the resulting challenges. More restrictions on the use of antibiotics are urgently needed from policy makers and authorities, more so than in the current situation. Combating AMR and achieving the global goals outlined in the SDGs requires coordination efforts on a global scale.

## Data Availability

All data generated or analysed during this study are included in this published article. In addition, other datasets used during the current study are available from the author on reasonable request (saedzyoud@yahoo.com).

## Abbreviations

COVID-19: Coronavirus disease 2019
SDGs: Sustainable development goals
AMR: Antimicrobial resistance
WHO: World health organization

## References

[1] Murray CJL, Ikuta KS, Sharara F, Swetschinski L, Robles Aguilar G, Gray A, Han C, Bisignano C, Rao P, Wool E (2022). Global burden of bacterial antimicrobial resistance in 2019: a systematic analysis. The Lancet.

[2] Prestinaci F, Pezzotti P, Pantosti A (2015). Antimicrobial resistance: a global multifaceted phenomenon. Pathog Glob Health.

[3] Zeshan B, Karobari MI, Afzal N, Siddiq A, Basha S, Basheer SN, Peeran SW, Mustafa M, Daud NHA, Ahmed N (2021). The usage of antibiotics by COVID-19 patients with comorbidities: the risk of increased antimicrobial resistance. Antibiotics.

[4] Ventola CL (2015). The antibiotic resistance crisis: part 1: causes and threats. P T.

[5] Popp M, Stegemann M, Riemer M, Metzendorf MI, Romero CS, Mikolajewska A, Kranke P, Meybohm P, Skoetz N, Weibel S (2021). Antibiotics for the treatment of COVID-19. Cochrane Database Syst Rev.

[6] Townsend L, Hughes G, Kerr C, Kelly M, O'Connor R, Sweeney E, Doyle C, O'Riordan R, Bergin C, Bannan C (2020). Bacterial pneumonia coinfection and antimicrobial therapy duration in SARS-CoV-2 (COVID-19) infection. JAC Antimicrob Resist.

[7] Thapa B, Pathak SB, Jha N, Sijapati MJ, Shankar PR (2022). Antibiotics use in hospitalised COVID-19 patients in a tertiary care centre: a descriptive cross-sectional study. JNMA J Nepal Med Assoc.

[8] Centers for disease control and prevention. COVID-19 & antimicrobial resistance. 2022. https://www.cdc.gov/drugresistance/covid19.html#:~:text=Antibiotics%20were%20commonly%20prescribed%20to,one%20that%20causes%20COVID%2D19. (Accessed Dec 9 2022).

[9] Jasovský D, Littmann J, Zorzet A, Cars O (2016). Antimicrobial resistance-a threat to the world's sustainable development. Ups J Med Sci.

[10] World Health Organization. Antimicrobial resistance. 2021. https://www.who.int/news-room/fact-sheets/detail/antimicrobial-resistance (Accessed Dec 9 2022).

[11] Xavier-Santos D, Padilha M, Fabiano GA, Vinderola G, Gomes Cruz A, Sivieri K, Costa Antunes AE (2022). Evidences and perspectives of the use of probiotics, prebiotics, synbiotics, and postbiotics as adjuvants for prevention and treatment of COVID-19: a bibliometric analysis and systematic review. Trends Food Sci Technol.

[12] Zyoud SH, Zyoud AH (2021). Coronavirus disease-19 in environmental fields: a bibliometric and visualization mapping analysis. Environ Dev Sustain.

[13] Mayta-Tovalino F (2022). Bibliometric analyses of global scholarly output in dentistry related to COVID-19. J Int Soc Prev Community Dent.

[14] Chan KIP, Ignacio KHD, Omar AT, Khu KJO (2022). Top 100 most cited neurologic and neurosurgical articles on COVID-19: a bibliometric analysis. World Neurosurg.

[15] Zhang Y, Hu M, Wang J, Wang P, Shi P, Zhao W, Liu X, Peng Q, Meng B, Feng X (2022). A bibliometric analysis of personal protective equipment and COVID-19 researches. Front Public Health.

[16] Otávio José de O, Fabio Francisco da S, Fernando J, Luis César Ferreira Motta B, Thaís Vieira N: Bibliometric method for mapping the state-of-the-art and identifying research gaps and trends in literature: an essential instrument to support the development of scientific projects. In: Scientometrics recent advances. edn. Edited by Suad K, Enver Z. Rijeka: IntechOpen; 2019: Ch. 3

[17] Ellegaard O, Wallin JA (2015). The bibliometric analysis of scholarly production: How great is the impact?. Scientometrics.

[18] Drew CH, Pettibone KG, Finch FO, Giles D, Jordan P (2016). Automated research impact assessment: a new bibliometrics approach. Scientometrics.

[19] Al-Jabi SW (2019). Arab world's growing contribution to global leishmaniasis research (1998–2017): a bibliometric study. BMC Public Health.

[20] Al-Jabi SW (2021). Current global research landscape on COVID-19 and depressive disorders: bibliometric and visualization analysis. World J Psychiatry.

[21] Sweileh WM (2022). Global research activity on mathematical modeling of transmission and control of 23 selected infectious disease outbreak. Global Health.

[22] Sweileh WM (2022). Patient satisfaction with nursing care: a bibliometric and visualization analysis (1950–2021). Int J Nurs Pract.

[23] Cabanillas-Lazo M, Quispe-Vicuna C, Barja-Ore J, Fernandez-Giusti A, Munive-Degregori A, Retamozo-Siancas Y, Guerrero ME, Mayta-Tovalino F (2022). A 10-year bibliometric analysis of global research on gut microbiota and parkinson's disease: characteristics, impact, and trends. Biomed Res Int.

[24] Bakkalbasi N, Bauer K, Glover J, Wang L (2006). Three options for citation tracking: google scholar, scopus and web of science. Biomed Digit Libr.

[25] Falagas ME, Pitsouni EI, Malietzis GA, Pappas G (2008). Comparison of PubMed, Scopus, web of science, and google scholar: strengths and weaknesses. FASEB J.

[26] Kulkarni AV, Aziz B, Shams I, Busse JW (2009). Comparisons of citations in Web of Science, Scopus, and Google Scholar for articles published in general medical journals. JAMA.

[27] Zyoud SH, Shakhshir M, Koni A, Shahwan M, Jairoun AA, Al-Jabi SW (2022). Olfactory and gustatory dysfunction in COVID-19: a global bibliometric and visualized analysis. Ann Otol Rhinol Laryngol.

[28] Zyoud SH, Al-Jabi SW (2020). Mapping the situation of research on coronavirus disease-19 (COVID-19): a preliminary bibliometric analysis during the early stage of the outbreak. BMC Infect Dis.

[29] Zyoud SH, Al-Jabi SW, Koni A, Shakhshir M, Shahwan M, Jairoun AA (2022). Mapping the landscape and structure of global research on nutrition and COVID-19: visualization analysis. J Health Popul Nutr.

[30] Zyoud SH, Koni A, Al-Jabi SW, Amer R, Shakhshir M, Al Subu R, Salameh H, Odeh R, Musleh S, Abushamma F (2022). Current global research landscape on COVID-19 and cancer: bibliometric and visualization analysis. World J Clin Oncol.

[31] Zyoud SH, Al-Jabi SW, Shahwan MJ, Jairoun AA (2022). Global research production pertaining to gastrointestinal involvement in COVID-19: a bibliometric and visualised study. World J Gastrointest Surg.

[32] Sweileh WM, Wickramage K, Pottie K, Hui C, Roberts B, Sawalha AF, Zyoud SH (2018). Bibliometric analysis of global migration health research in peer-reviewed literature (2000–2016). BMC Public Health.

[33] Lastella M, Memon AR, Vincent GE (2020). Global research output on sleep research in athletes from 1966 to 2019: a bibliometric analysis. Clocks Sleep.

[34] Sweileh WM, Huijer HA, Al-Jabi SW, Zyoud SH, Sawalha AF (2019). Nursing and midwifery research activity in Arab countries from 1950 to 2017. BMC Health Serv Res.

[35] Karasneh RA, Al-Azzam SI, Alzoubi KH, Hawamdeh SS, Sweileh WM (2022). Global research trends of health-related publications on ramadan fasting from 1999 to 2021: a bibliometric analysis. J Relig Health.

[36] Sweileh WM (2021). Substandard and falsified medical products: bibliometric analysis and mapping of scientific research. Global Health.

[37] van Eck NJ, Waltman L (2010). Software survey: VOSviewer, a computer program for bibliometric mapping. Scientometrics.

[38] Imani SM, Ladouceur L, Marshall T, Maclachlan R, Soleymani L, Didar TF (2020). Antimicrobial nanomaterials and coatings: current mechanisms and future perspectives to control the spread of viruses including SARS-CoV-2. ACS Nano.

[39] Hsu J (2020). How covid-19 is accelerating the threat of antimicrobial resistance. BMJ.

[40] Getahun H, Smith I, Trivedi K, Paulin S, Balkhy HH (2020). Tackling antimicrobial resistance in the COVID-19 pandemic. Bull World Health Organ.

[41] Clancy CJ, Nguyen MH (2020). Coronavirus disease 2019, superinfections, and antimicrobial development: what can we expect?. Clin Infect Dis.

[42] Rawson TM, Moore LSP, Zhu N, Ranganathan N, Skolimowska K, Gilchrist M, Satta G, Cooke G, Holmes A (2020). Bacterial and fungal coinfection in individuals with Coronavirus: a rapid review to support COVID-19 antimicrobial prescribing. Clin Infect Dis.

[43] Vaughn VM, Gandhi TN, Petty LA, Patel PK, Prescott HC, Malani AN, Ratz D, McLaughlin E, Chopra V, Flanders SA (2021). Empiric antibacterial therapy and community-onset bacterial coinfection in patients hospitalized with Coronavirus disease 2019 (COVID-19): a multi-hospital cohort study. Clin Infect Dis.

[44] Huttner BD, Catho G, Pano-Pardo JR, Pulcini C, Schouten J (2020). COVID-19: don't neglect antimicrobial stewardship principles!. Clin Microbiol Infect.

[45] Langford BJ, So M, Raybardhan S, Leung V, Soucy JR, Westwood D, Daneman N, MacFadden DR (2021). Antibiotic prescribing in patients with COVID-19: rapid review and meta-analysis. Clin Microbiol Infect.

[46] Beović B, Doušak M, Ferreira-Coimbra J, Nadrah K, Rubulotta F, Belliato M, Berger-Estilita J, Ayoade F, Rello J, Erdem H (2020). Antibiotic use in patients with COVID-19: a 'snapshot' infectious diseases international research initiative (ID-IRI) survey. J Antimicrob Chemother.

[47] Rawson TM, Moore LSP, Castro-Sanchez E, Charani E, Davies F, Satta G, Ellington MJ, Holmes AH (2020). COVID-19 and the potential long-term impact on antimicrobial resistance. J Antimicrob Chemother.

[48] Sweileh WM, Moh'd Mansour A (2020). Bibliometric analysis of global research output on antimicrobial resistance in the environment (2000–2019). Glob Health Res Policy.

[49] Farooq RK, Rehman SU, Ashiq M, Siddique N, Ahmad S (2021). Bibliometric analysis of coronavirus disease (COVID-19) literature published in Web of Science 2019–2020. J Family Community Med.

[50] Qu J, Huang Y, Lv X (2019). Crisis of antimicrobial resistance in China: now and the future. Front Microbiol.

[51] Hu F, Zhu D, Wang F, Wang M (2018). Current status and trends of antibacterial resistance in China. Clin Infect Dis.

[52] Van Boeckel TP, Pires J, Silvester R, Zhao C, Song J, Criscuolo NG, Gilbert M, Bonhoeffer S, Laxminarayan R (2019). Global trends in antimicrobial resistance in animals in low- and middle-income countries. Science.

[53] Jacob BA, Lefgren L (2011). The impact of research grant funding on scientific productivity. J Public Econ.

[54] Neema S, Chandrashekar L (2021). Research funding-why, when, and how?. Indian Dermatol Online J.

[55] Granata G, Schiavone F, Pipitone G, Taglietti F, Petrosillo N (2022). Antibiotics use in COVID-19 patients: a systematic literature review. J Clin Med.

[56] Pierce J, Stevens MP (2021). COVID-19 and antimicrobial stewardship: lessons learned, best practices, and future implications. Int J Infect Dis.

[57] Sweileh WM (2021). Bibliometric analysis of peer-reviewed literature on antimicrobial stewardship from 1990 to 2019. Global Health.

[58] Sweileh WM (2021). Global research publications on irrational use of antimicrobials: call for more research to contain antimicrobial resistance. Global Health.

[59] Rusic D, Vilovic M, Bukic J, Leskur D, Seselja Perisin A, Kumric M, Martinovic D, Petric A, Modun D, Bozic J (2021). Implications of COVID-19 Pandemic on the emergence of antimicrobial resistance: adjusting the response to future outbreaks. Life (Basel).

[60] Sulayyim HJA, Ismail R, Hamid AA, Ghafar NA (2022). Antibiotic resistance during COVID-19: a systematic review. Int J Environ Res Public Health.

